# Clinical and laboratory aspects of patients diagnosed with various inherited platelet disorders

**DOI:** 10.1016/j.rpth.2025.102873

**Published:** 2025-04-24

**Authors:** Veysel Gök, Alper Ozcan, Fatma Türkan Mutlu, Ebru Yılmaz, Deniz Kocak Göl, Mustafa Ozay, Baver Demir, Hüseyin Taskiran, Hasan Bas, Mehmet Burak Mutlu, Muhammet Ensar Dogan, Atil Bisgin, Ido Somekh, Meino Rohlfs, Munis Dundar, Yusuf Ozkul, Christoph Klein, Musa Karakukcu, Ekrem Unal

**Affiliations:** 1 Division of Pediatric Hematology and Oncology, Department of Pediatrics, School of Medicine, Erciyes University, Kayseri, Türkiye; 2 Division of Pediatric Hematology and Oncology, Department of Pediatrics, Kayseri City Hospital, Kayseri, Türkiye; 3 Department of Pediatric Hematology and Oncology, Gaziantep City Hospital, Gaziantep, Türkiye; 4 Department of Pediatric Hematology and Oncology, Medical Point Hospital, Gaziantep, Türkiye; 5 Department of Internal Medicine, Medical Point Hospital, Gaziantep, Türkiye; 6 Intergen Genetics and Rare Diseases Diagnosis Center, Ankara, Türkiye; 7 DETAGEN Genetic Diseases Evaluation Center, Kayseri, Türkiye; 8 Department of Medical Genetics, Kayseri City Hospital, Kayseri, Türkiye; 9 Department of Medical Genetics, School of Medicine, Çukurova University, Adana, Türkiye; 10 Pediatric Department A and the Immunology Service, Sheba Medical Center, Tel Aviv University, Tel Aviv, Israel; 11 Division of Pediatric Hematology and Oncology, Department of Pediatrics, Dr von Hauner Children’s Hospital, Ludwig Maximilians University, Munich, Germany; 12 Department of Medical Genetics, School of Medicine, Erciyes University, Kayseri, Türkiye; 13 Department of Nursing, School of Health Sciences, Hasan Kalyoncu University, Gaziantep, Türkiye

**Keywords:** genetics, hematopoietic stem cell transplantation, inherited platelet disorders, platelet count, thrombocytopenia

## Abstract

**Background:**

Inherited platelet disorders (IPDs) are characterized by thrombocytopenia, platelet dysfunction, or both, leading to recurrent bleeding and diagnostic challenges. Advances in genetic testing have significantly improved early and accurate diagnoses.

**Objectives:**

This study aimed to evaluate the clinical and genetic spectrum of IPDs, identify diagnostic challenges, and assess outcomes of therapeutic interventions.

**Methods:**

We conducted a retrospective cohort study of 50 IPD patients. We performed clinical evaluations, peripheral smear analyses, and genetic testing to identify causative variants. Correlation between platelet counts, bleeding severity, and the effectiveness of treatments, such as hematopoietic stem cell transplantation and thrombopoietin receptor agonists, was analyzed.

**Results:**

A total of 54.5% of cases showed autosomal dominant inheritance. Diagnostic delays were common, with many patients initially misdiagnosed as having immune thrombocytopenic purpura (ITP). There was a moderate, negative, statistically significant correlation between platelet counts and bleeding severity. Peripheral smear findings, such as stomatocytosis and macrothrombocytopenia, provided critical diagnostic clues. We identified novel mutations in *GP1BA*, *ITGB3*, *NBEAL2*, *WAS*, and *MPL* genes, which expanded our understanding of IPDs. Different treatment modalities were used. Hematopoietic stem cell transplantation was performed in severe systemic cases, such as Wiskott–Aldrich syndrome. Sitosterolemia was treated with ezetimibe. Thrombopoietin receptor agonists reduced bleeding in some patients.

**Conclusion:**

Integrating genetic, clinical, and laboratory findings is essential in providing accurate diagnoses and management of IPDs. Early genetic diagnosis and personalized therapeutic strategies improve outcomes. Future research should focus on functional studies of novel mutations and refining treatment protocols to enhance care for this complex population.

## Essentials

• Inherited platelet disorders are genetically diverse conditions that cause low platelet count, platelet dysfunction, or both, often leading to bleeding and delayed diagnosis.
• This study retrospectively evaluated 50 pediatric patients using clinical findings, peripheral smear morphology, and whole-exome sequencing across multiple centers in Türkiye.
• Genetic testing revealed several novel and rare disease-related variants, expanding the mutational spectrum of inherited platelet disorders.
• Integrating clinical and genetic data improved diagnostic accuracy and supported more personalized treatment strategies for affected patients.

## 1 Introduction

Inherited platelet disorders (IPDs) encompass a heterogeneous group of rare genetic conditions characterized by abnormalities in platelet number, function, or both. These disorders predispose individuals to varying degrees of bleeding, typically involving the skin and mucosal surfaces. Clinical manifestations commonly include petechiae, purpura, epistaxis, menorrhagia, gastrointestinal bleeding, and, in severe cases, central nervous system (CNS) hemorrhages [[1], [2], [3]].

The exact prevalence of IPDs remains uncertain due to diagnostic challenges and phenotypic variability; however, estimates suggest a range between 1 per 10,000 and 1 per 1,000,000 individuals [1,2]. Diagnostic difficulties primarily arise from clinical heterogeneity, overlapping symptoms with acquired disorders such as immune thrombocytopenic purpura (ITP), and limited availability of specialized diagnostic techniques and guidelines [4,5].

IPDs are genetically diverse, involving mutations in approximately 75 genes associated with platelet production, function, or both [6]. These disorders can be inherited via autosomal dominant, autosomal recessive, or X-linked recessive patterns. Clinical severity varies widely, even among individuals harboring identical mutations, highlighting the complexity of genotype–phenotype correlations [[7], [8], [9]].

Accurate and timely diagnosis of IPDs is crucial, as misdiagnosis can lead to inappropriate interventions such as unnecessary splenectomies, ineffective treatments, and a delay in proper management. Recent advances in genetic technologies, particularly next-generation sequencing, have significantly improved the ability to identify pathogenic genetic variants, thus enhancing diagnostic precision and allowing for individualized management strategies [[10], [11], [12]].

In Türkiye, increased consanguinity rates contribute to a distinctive genetic landscape, with a higher incidence of autosomal recessive conditions, including IPDs. This demographic characteristic underscores the need for comprehensive genetic evaluations and tailored clinical management approaches in affected populations [13,14].

This study aimed to delineate the clinical and genetic spectrum of IPDs within a cohort of Turkish patients, emphasizing the diagnostic challenges, genotype–phenotype correlations, and outcomes of various therapeutic strategies. We anticipate that our findings will contribute to a better understanding of IPDs and promote improvements in diagnosis, management, and overall patient care.

## 2 Methods

### 2.1 Patients and ethical approval

In this retrospective cohort study, 5 pediatric hematology centers in Türkiye—Erciyes University (Kayseri), Kayseri City Hospital (Kayseri), Atatürk University (Erzurum), Gaziantep City Hospital (Gaziantep), and Medical Point Hospital (Gaziantep)—evaluated patients diagnosed with thrombocytopenia and/or platelet function disorders for potential IPDs. Hospital electronic medical records and patient files of 50 patients with recognized genetic variations linked to IPDs were analyzed retrospectively. Data collected included initial presentation age, symptoms, bleeding severity, pregenetic diagnosis, treatments administered, implicated genes and mutations, and supplementary findings such as nonhematological characteristics. Written informed consent was explicitly obtained from patients or their legal guardians at each participating center, specifically including permission for genetic testing and retrospective analysis of clinical data for research purposes. The study received approval from the Erciyes University Ethics Committee (approval number: 2023/357). This comprehensive analysis emphasizes the genetic and clinical diversity of IPDs in regions with higher consanguinity rates.

### 2.2 Clinical bleeding and severity

We carefully documented clinical hemorrhagic manifestations at diagnosis and during follow-up using structured clinical evaluation tools. Given the multicenter and retrospective design of this study, a simplified structured clinical evaluation form was specifically developed and utilized across participating centers. This form included clearly defined fields to uniformly document onset, duration, triggers, anatomical sites, and severity grading of bleeding episodes. Although the International Society on Thrombosis and Haemostasis (ISTH) Bleeding Assessment Tool represents the gold standard for bleeding assessment in IPDs, our pragmatic approach facilitated consistent and efficient data extraction from retrospective medical records, ensuring practicality and uniformity in a multicenter setting [15,16]. The forms contained designated fields to document the onset, length, and triggers of bleeding episodes, assuring a uniform method of data collection and facilitating replication. Life-threatening hemorrhagic events, such as gastrointestinal or CNS bleeds, were documented individually. These events were characterized by the necessity for prompt medical intervention, blood transfusion, or leading to extended hospitalization or lifelong disability. Bleeding episodes were categorized into 4 classifications according to the European Network of Rare Bleeding Disorders guidelines: asymptomatic (absence of bleeding symptoms despite low platelet counts), trauma-related (bleeding induced by external injuries), and spontaneous minor (bleeding events like mild epistaxis or minor bruising without identifiable cause) or major bleeding (severe incidents leading to medical intervention, such as gastrointestinal or CNS hemorrhages) [17].

### 2.3 Statistical analysis

Statistical analyses were performed using the TURCOSA Analytics program. Spearman’s correlation coefficient was employed separately to assess the association between platelet counts and bleeding severity in 2 distinct patient groups: inherited platelet function disorders (IPFD) and isolated thrombocytopenias (IT). Odds ratios were calculated to determine the risk of life-threatening bleeding episodes in patients with platelet counts below 10 × 10^3^/mm^3^. A *P* value of < .05 was considered statistically significant. This differentiated analysis facilitated a clearer interpretation of how platelet numbers specifically impact bleeding severity within distinct IPD subtypes.

### 2.4 Blood cell counts and peripheral blood smear examinations

We analyzed the complete blood count results and peripheral blood smears for each patient. We reviewed peripheral blood smears at least 14 days after the last platelet transfusion to ensure an accurate evaluation of platelet morphology and count. Peripheral blood smears evaluated in this study included initial diagnostic smears and, when necessary, subsequent follow-up smears. Specifically, for patients who received platelet transfusions or had diagnostic uncertainties at initial presentation, smears were reexamined at least 14 days after the last platelet transfusion. This approach ensured the accurate assessment of platelet morphology and counts without transfusion-related alterations, thus providing reliable diagnostic insights across the patient cohort. Key parameters included platelet count, hemoglobin, mean platelet volume (MPV), and morphologic findings of platelets, erythrocytes, and leukocytes. Thresholds for anemia were defined according to World Health Organization guidelines as follows: 11 g/dL for 0.50 to 4.99 years, 11.5 g/dL for 5 to 11.99 years, 12 g/dL for 12 to 14.99 years, 12 g/dL for women, and 13 g/dL for men. MPV reference values ranged from 7.2 to 11.7 fL [18]. Smears specifically noted abnormalities such as macrothrombocytes, microthrombocytes, or other atypical findings.

### 2.5 Platelet function testing

Platelet function testing was performed in a subset of patients (*n* = 9) who presented with bleeding phenotypes and peripheral smear findings suggestive of Bernard–Soulier syndrome (BSS), Glanzmann thrombasthenia (GT), or Gray platelet syndrome. The tests included light transmission aggregometry (LTA) using standard agonists (adenosine diphosphate [ADP], collagen, and ristocetin) and flow cytometry for platelet surface glycoprotein (GP) expression (eg, CD41 and CD42b). Functional test results were interpreted in conjunction with clinical and genetic data to support diagnostic classification.

### 2.6 Genetic analysis

Multiple accredited laboratories performed genetic testing using next-generation sequencing methods to investigate variants implicated in platelet disorders. Two principal strategies were employed: (1) targeted gene panel testing for known platelet disorder genes in certain patients and (2) comprehensive whole-exome sequencing for others, aiming to uncover novel or less common variants.

Although the participating laboratories used different platforms and library preparation kits, a standardized approach to quality control ensured consistent minimum coverage thresholds (eg, ≥20× for >95% of target regions). When an initial variant of clinical significance was suspected, confirmatory Sanger sequencing or alternative validation methods were performed. For frameshift or nonsense variants, we evaluated the likelihood of nonsense-mediated mRNA decay, incorporating patient phenotype data to support pathogenicity. Where possible, we also performed familial segregation analyses to enhance variant interpretation, particularly in autosomal dominant genes.

All identified variants were re-evaluated with reference to current scientific databases (eg, Clinical Variants Database [ClinVar], Genome Aggregation Database [gnomAD]) and literature to assess their novelty, frequency, and known disease associations. While the American College of Medical Genetics and Genomics (ACMG) and Clinical Genome Resource (ClinGen) guidelines served as a foundational framework for classification, we adapted certain criteria—such as pathogenic strong criterion 3 (PS3, functional data) and pathogenic supporting criterion 1 (PP1, family segregation)—to match the retrospective nature of the study and to account for each variant’s molecular characteristics. In situations where complete familial data or functional assays were unavailable, *in silico* predictions and the patient’s clinical presentation were integrated to infer pathogenicity.

Finally, we correlated genetic findings with clinical and laboratory parameters (eg, platelet count and morphology, bleeding severity, and additional phenotypic features) to validate plausibility. For novel frameshift variants or variants of uncertain significance (VUS), multidisciplinary team meetings were held to reach consensus on their clinical relevance based on the totality of evidence—genetic, clinical, and, in certain instances, familial segregation.

### 2.7 Bioinformatics of exome sequencing

Standard bioinformatics pipelines processed the exome sequencing data, performing quality control, alignment, variant calling, and annotation. We prioritized variants based on their novelty, inheritance pattern, and association with genes related to platelets. We assessed pathogenicity using computational tools, population databases, and literature review. Multidisciplinary team meetings discussed VUS to establish a consensus on their clinical relevance. Novel variants underwent *in silico* functional analysis to predict their impact on protein function.

Exome sequencing data underwent standardized bioinformatic processing, including quality trimming, sequence alignment to the human reference genome (GRCh37 or GRCh38, depending on the laboratory), variant calling, and annotation. Average exome coverage, variant read depth, and on-target rates met or exceeded established laboratory benchmarks. Variant filtering prioritized rare (minor allele frequency <1%) and protein-altering changes relevant to thrombocytopenia or platelet function.

Pathogenicity assessments integrated multiple lines of evidence, including computational prediction tools such as SIFT (developed at the Fred Hutchinson Cancer Research Center), PolyPhen-2 (developed at Harvard Medical School), and MutationTaster (developed at Charité–Universitätsmedizin Berlin), in addition to gene-specific knowledge bases. However, for novel frameshift or nonsense variants, *in silico* tools were used cautiously, as they are typically designed for evaluating missense variants. Instead, we employed additional criteria such as presumed loss-of-function effects, nonsense-mediated decay pathways, and patient-specific phenotype–genotype correlations. Multidisciplinary teams, comprising clinical geneticists, pediatric hematologists, and molecular biologists, convened to discuss variants classified as VUS and interpret them in light of clinical severity, family history, and any additional laboratory findings.

## 3 Results

### 3.1 Demographic and clinical data

The cohort consisted of 30 males and 20 females, with a median current age of 10.36 years (range, 0.4-37.02; Table 1). Consanguinity was present in 29 of 50 patients (58%), reflecting regional patterns. Only 14 patients (28%) had a family history of bleeding diathesis. The median age at initial presentation was 0.67 years (range, 0-34.7 years), and the median age at genetic diagnosis was 3.41 years (range, 2 months to 35.9 years). The median time from presentation to diagnosis was 1.25 years (range, 1 day to 21.02 years). Before diagnosis, the patients had the following conditions: chronic ITP (20 patients, 40%), thrombocytopenia of unknown cause (19 patients, 38%), neonatal alloimmune thrombocytopenia (4 patients, 8%), immunodeficiency (2 patients, 4%), pancytopenia (2 patients, 4%), neutropenia (2 patients, 4%), and anemia (1 patient, 2%). A total of 38 patients (76%) had incompatible pre- and postgenetic diagnoses. Additionally, at some point in their lives, 38 of 50 patients (76%) received platelet transfusions, and 40 patients (80%) had anemia. Four patients died due to infections following transplantation, while 3 patients succumbed to severe bleeding episodes. We noted a diverse range of clinical presentations, including bleeding and nonhematological symptoms.

**Table 1:** Clinical and bleeding features of 50 patients diagnosed with inherited platelet disorder.

Patient	Gender	Responsible gene	Age at admission	Admission symptoms	Pregenetic disorder	Age at diagnosis	Most frequent bleeding sites	Bleeding severity (grade)	Life-threatening bleeding	Anemia (yes/no)	Additional findings	Treatment	Outcome
1	M	*ABCG5*	7 y	Easy bruising	Thrombocytopenia	9 y	Skin	1	No	Yes	Hemolytic anemia	Ezetimibe	Alive
2	F	*ABCG5*	8 y	Pallor	Anemia	9 y	Skin	1	No	Yes	Hemolytic anemia, epilepsy, and glaucoma	Ezetimibe	Alive
3	M	*ETV6*	3 mo	Petechiae	Chronic ITP	18 mo	Skin	1	No	Yes	None	None	Alive
4	F	*ETV6*	9 y	Abdominal pain, recurrent infection	Neutropenia	14 y	None	0	No	No	Hypogammaglobinemia, asthma	IVIG	Alive
5	M	*FYB1*	9 mo	Neutropenia	Neutropenia	17 mo	None	0	No	No	None	None	Alive
6	F	*MYH9*	10 d	Easy bruising	NAIT	6 mo	Skin	1	No	Yes	None	Platelets	Alive
7	F	*MYH9*	2 mo	Easy bruising, petechiae	Chronic ITP	17 mo	Skin	2	No	Yes	None	Platelets	Alive
8	M	*MYH9*	8 y	Easy bruising, epistaxis	Thrombocytopenia	9 y	Skin, nose	1	No	No	None	Tranexamic acid	Alive
9	F	*MYH9*	34 y	Easy bruising, epistaxis	Thrombocytopenia	36 y	Skin, nose	1	No	No	None	Tranexamic acid	Alive
10	M	*MYH9*	10 y	Easy bruising	Thrombocytopenia	20 y	Skin	1	No	No	None	None	Alive
11	F	*GP1BA*	7 y	Epistaxis	Chronic ITP	13 y	Skin, nose, and uterine	3	GIS	Yes	None	Platelets, tranexamic acid	Alive
12	M	*GP1BA*	11 mo	Easy bruising, epistaxis	Chronic ITP	2 y	Skin, mouth, and nose	2	No	Yes	None	Platelets, tranexamic acid	Alive
13	F	*GP1BA*	3 y	Easy bruising, epistaxis	Chronic ITP	14 y	Skin, mouth, and uterine	2	No	Yes	Autism, mental retardation	Platelets, tranexamic acid	Alive
14	M	*GP1BA*	8 mo	Easy bruising, petechiae	Chronic ITP	10 y	Nose, skin, and GIS	3	GIS	Yes	None	Platelets, tranexamic acid	Alive
15	F	*ITGB3*	11 y	Easy bruising	Chronic ITP	16 y	Skin, uterine	2	No	Yes	None	Platelets, tranexamic acid	Alive
16	F	*ITGB3*	1.5 y	Easy bruising, petechiae, and epistaxis	Thrombocytopenia	18 y	Skin, nose	2	No	Yes	None	Platelets, tranexamic acid	Alive
17	M	*NBEAL2*	1.5 mo	Easy bruising	Chronic ITP	8 y	Skin	1	No	No	None	Platelets	Alive
18	M	*NBEAL2*	2 y	Easy bruising, epistaxis	Chronic ITP	10 y	Nose, skin	1	No	No	None	Platelets	Alive
19	F	*NBEAL2*	9 y	Abdominal pain, easy bruising	Chronic ITP	10 y	None	0	No	No	None	Platelets	Alive
20	F	*WAS*	14 mo	Easy bruising, epistaxis	Chronic ITP	6 y	Skin, mouth, and nose	2	No	Yes	Eczema, recurrent otitis	Platelets, HSCT	Alive
21	M	*WAS*	18 mo	Easy bruising, petechiae, and eczema	Chronic ITP	11 y	Skin	2	No	Yes	Aortic coarctation, brain abscess, frequent pneumonia, and eczema	Platelets, HSCT	Alive
22	M	*WAS*	13 mo	Easy bruising, petechiae, eczema, and recurrent infection	Thrombocytopenia	9 y	Skin, nose	2	No	Yes	Frequent pneumonia, eczema	Platelets, HSCT	Alive
23	M	*WAS*	3 mo	Coughing, fever, petechiae, eczema, and blood in stool	Thrombocytopenia	4 mo	Skin, GIS, and CNS	3	CNS	Yes	Frequent infections, eczema	Platelets	Deceased
24	M	*WAS*	4 mo	Blood in stool	Thrombocytopenia	8 mo	Skin, CNS	3	CNS	Yes	Frequent infections, eczema	Platelets	Deceased
25	M	*WAS*	4 d	Petechiae	Thrombocytopenia	2 mo	Skin	2	No	Yes	Frequent infections, eczema	Platelets, HSCT	Alive
26	M	*WAS*	6 mo	Easy bruising, petechiae	Thrombocytopenia	6 y	Skin	2	No	No	Eczema, frequent infections, and aortic aneurysm	Platelets	Alive
27	M	*WAS*	4 mo	Petechiae, eczema, and blood in urine	Thrombocytopenia	5 mo	Skin	2	No	Yes	Eczema	Platelets, HSCT	Alive
28	M	*WAS*	3 mo	Petechiae, eczema, and coughing	Immunodeficiency	5 mo	Skin, mouth, nose, and GIS	3	GIS	Yes	Eczema, frequent pneumonia	Platelets, HSCT	Alive
29	M	*WAS*	6 mo	Coughing, fever, petechiae, and eczema	Immunodeficiency	7 mo	Skin, mouth, nose, and GIS	3	GIS	Yes	Eczema, frequent pneumonia	Platelets, HSCT	Deceased
30	M	*WAS*	3 y	Bleeding in the oral cavity	Chronic ITP	15.5 y	Skin, mouth, and nose	2	No	Yes	Frequent pneumonia	Platelets, HSCT	Deceased
31	M	*WAS*	11 mo	Coughing, petechiae	Thrombocytopenia	5 y	Skin	2	No	Yes	Eczema, frequent infections	Platelets, HSCT	Alive
32	M	*WAS*	1 mo	Easy bruising, coughing, and fever	Thrombocytopenia	10 mo	Skin, nose	2	No	Yes	Eczema, frequent infections	Platelets, HSCT	Alive
33	M	*WAS*	11 d	Blood in stool	Thrombocytopenia	6 mo	Skin	2	No	Yes	Eczema, cow milk allergy	Platelets, HSCT	Alive
34	M	*WAS*	3 mo	Easy bruising, petechiae, and eczema	Thrombocytopenia	5 mo	Skin, nose	2	No	Yes	Eczema	Platelets, HSCT	Alive
35	M	*WAS*	2 mo	Petechiae, blood in stool	NAIT	3 mo	Skin, GIS	2	No	Yes	Cow milk allergy	Platelets, IVIG	Alive
36	F	*VWF*	4 d	Blood in stool	NAIT	7 d	Skin, GIS	3	GIS	Yes	None	Platelets, VWF concentrate	Alive
37	M	*GNE*	7 d	Easy bruising, petechiae	Thrombocytopenia	2 y	Skin, nose, and GIS	3	GIS	Yes	None	Platelets, HSCT	Deceased
38	M	*CDC42*	25 d	Sores in oral cavity, epistaxis, easy bruising, coughing, maculopapular rash, hypotonicity, and growth retardation	Thrombocytopenia	4.5 y	Skin, nose	1	No	Yes	Leukopenia, osteoporosis, hypotonic, and growth retardation	IVIG, platelets	Alive
39	M	*IKZF1*	3 mo	Easy bruising, petechiae, and pallor	Pancytopenia	6 mo	Skin, CNS	3	CNS	Yes	Pancytopenia	Platelets, erythrocytes, and HSCT	Deceased
40	F	*GFI1B*	40 d	Coughing, respiratory distress	Pancytopenia	10 mo	Skin, GIS	3	GIS	Yes	Immunodeficiency, frequent pneumonia	Platelets, IVIG	Deceased
41	F	*RBM8A*	Newborn	Short arm abnormality	Thrombocytopenia	3 d	Skin	2	No	Yes	Shortened/absent radii bilaterally	Platelets	Alive
42	F	*RBM8A*	15 d	Short arm abnormality, easy bruising	Thrombocytopenia	16 d	Skin, nose	2	No	Yes	Shortened/absent radii bilaterally	Platelets	Alive
43	F	*TUBB1*	16 mo	Easy bruising, epistaxis	Chronic ITP	22 y	Skin, mouth, and nose	2	No	Yes	None	Platelets	Alive
44	M	*RUNX1*	3 y	Easy bruising, epistaxis	Chronic ITP	4 y	Skin, nose	2	No	Yes	None	TPO-RAs	Alive
45	M	*FLI1*	5 mo	Easy bruising, petechiae, and bleeding in oral cavity	Chronic ITP	2 y	Skin	2	No	Yes	None	TPO-RAs	Alive
46	M	*ACTB*	8 mo	Easy bruising, petechiae	Chronic ITP	17.5 y	Skin, nose	1	No	No	Keratoconus	Platelets	Alive
47	F	*STIM1*	12 y	Easy bruising, petechiae	Chronic ITP	13 y	Skin, uterine	2	No	Yes	None	TPO-RAs	Alive
48	F	*MPL*	2 y	Easy bruising, petechiae, and epistaxis	Chronic ITP	3 y	Skin, nose	2	No	Yes	None	Platelets, TPO-RAs, and HSCT	Alive
49	F	*MPL*	3 d	Petechiae	NAIT	7 mo	Skin	2	No	Yes	None	Platelets, TPO-RAs, and HSCT	Alive
50	F	*ANKRD26*	7 y	Easy bruising, petechiae	Chronic ITP	13 y	Uterine	1	No	Yes	None	TPO-RAs	Alive

CNS, central nervous system; F, female; GIS, gastrointestinal system; HSCT, hematopoietic stem cell transplantation; ITP, immune thrombocytopenic purpura; IVIG, intravenous immunoglobulin; M, male; NAIT, neonatal alloimmune thrombocytopenia; TPO-RA, thrombopoietin receptor agonist; VWF, von Willebrand factor.

### 3.2 Bleeding episodes and disease-associated phenotypes

Petechiae and bruising were the most common initial symptoms (45.4%), followed by nose/mouth bleeding (15.6%), eczema (10.4%), infections (10.4%), and blood in stool (6.5%). Rare presentations included urinary tract bleeding (1.3%) and extremity anomalies (2.6%). Among 50 patients, 10 (20%) experienced life-threatening bleeding episodes, with gastrointestinal system (7 patients) and CNS (3 patients) hemorrhages being the most severe. Across the entire cohort, the most frequently affected bleeding sites were skin (48.3%), nose (23%), gastrointestinal tract (9.2%), mouth (6.9%), uterine (5.8%), CNS (3.4%), and other unspecified sites (3.4%; Figure). We categorized the bleeding severity as asymptomatic (*n* = 3, 6%), grade I bleeding (*n* = 12, 24%), grade II bleeding (*n* = 25, 50%), and grade III bleeding (*n* = 10, 20%). These findings highlight the critical importance of early diagnosis and intervention.

**Figure:**
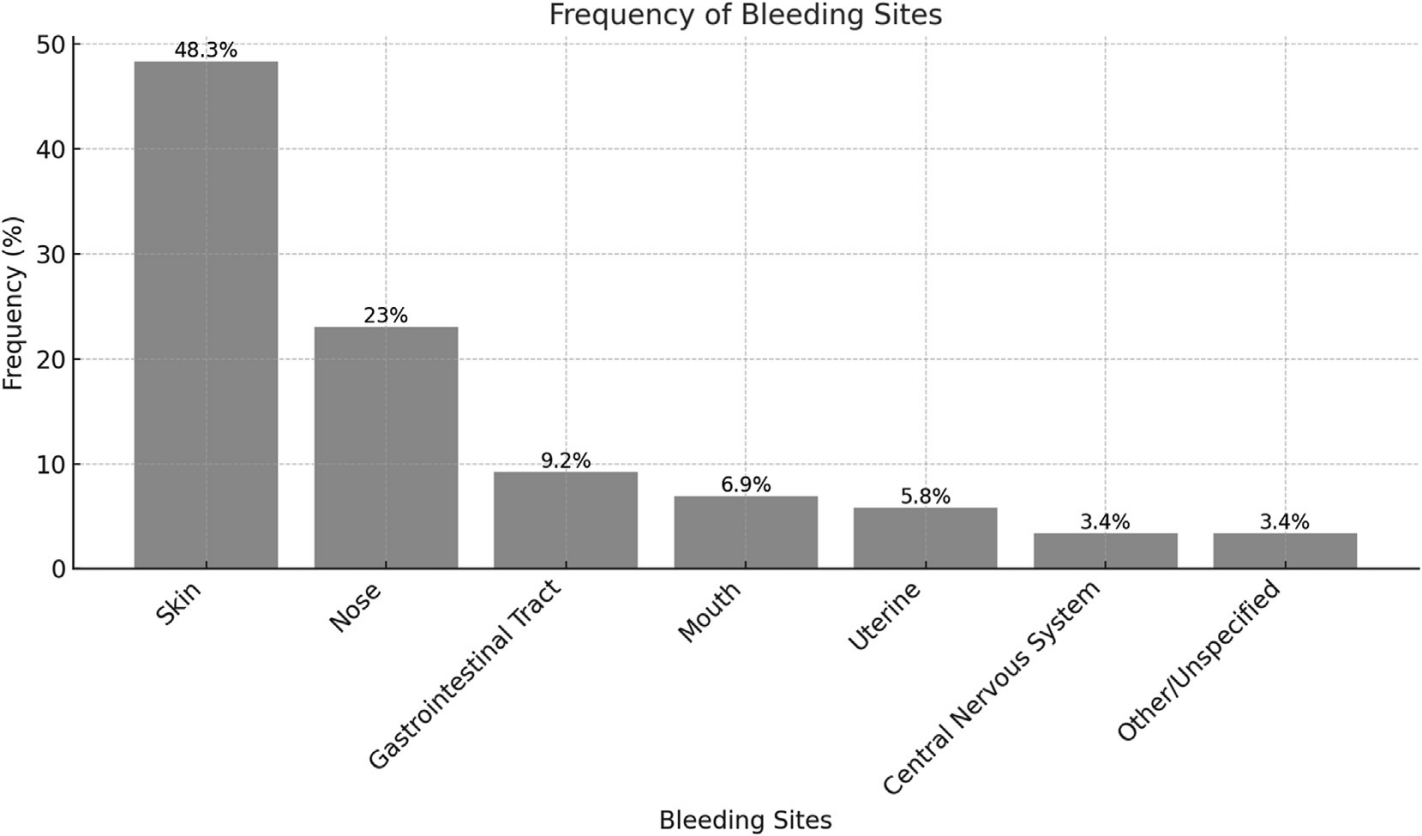
Frequency of bleeding sites of patients with inherited platelet disorders.

Disease-associated phenotypes such as eczema and recurrent infections were observed almost exclusively in WAS patients, affecting approximately 95% and 90%, respectively. Additionally, a patient with a *GFI1B* variant also exhibited recurrent infections. These findings underline the importance of considering syndromic features alongside bleeding symptoms when evaluating IPDs.

### 3.3 Basic laboratory results, platelet count, and morphology

Platelet counts exhibited significant variability, with median lowest and highest values of 8 × 10^3^/mm^3^ (range, 1-189) and 60.5 × 10^3^/mm^3^ (range, 18-431), respectively. Peripheral smear analysis revealed macrothrombocytes in 19 patients, microthrombocytes in 16 patients, and normothrombocytes in 15 patients. Patients with *WAS* mutations exclusively exhibited microthrombocytes. Additionally, peripheral smears of 2 patients with *ABCG5* mutations showed stomatocytosis and hemolysis alongside macrothrombocytes. All patients with microthrombocytes on peripheral smears were diagnosed with *WAS*, with a median MPV value of 6.85 fL (range, 4.8-8.8). In contrast, patients with macrothrombocytes had a median MPV value of 12.9 fL (range, 11.9-18). These morphologic findings correlated strongly with specific genetic variants (Table 2). Furthermore, 40 of 50 patients (80%) had anemia, with a median hemoglobin level of 10.3 g/dL (range, 5.3-14.8).

**Table 2:** Distribution of inherited platelet disorders according to platelet size, platelet count, and peripheral blood smear.

Platelet size	Patient and gender	Responsible gene	Disease definition and inheritance	Lowest Plt count (×10^3^/mm^3^)	Highest Plt count (×10^3^/mm^3^)	MPV (fL; 7.2-11.7)	Peripheral blood smear
**Macrothrombocyte**	1, M	*ABCG5*	Sitosterolemia, AR	61	97	15	Macrothrombocyte, schistocyte, and stomatocytes
	2, F	*ABCG5*		99	205	12.5	Macrothrombocyte, schistocyte, and stomatocytes
	6, F	*MYH9*	Macrothrombocytopenia and granulocyte inclusions with or without nephritis or sensorineural hearing loss, AD	4	20	12.9	Macrothrombocyte, cytoplasmic inclusion bodies within leukocytes
	7, F	*MYH9*		16	58	13.5	Macrothrombocyte
	8, M	*MYH9*		19	45	12	Macrothrombocyte
	9, F	*MYH9*		14	54	13	Macrothrombocyte
	10, M	*MYH9*		48	83	12	Macrothrombocyte, cytoplasmic inclusion bodies within leukocytes
	11, F	*GP1BA*	BSS, AR	24	32	12	Macrothrombocyte
	12, M	*GP1BA*		14	60	18	Macrothrombocyte
	13, F	*GP1BA*		9	78	15.1	Macrothrombocyte
	14, M	*GP1BA*		10	54	13.5	Macrothrombocyte
	15, F	*ITGB3*	Platelet-type 24, bleeding disorder, AD	15	126	12.7	Macrothrombocyte
	17, M	*NBEAL2*	Gray platelet syndrome, AR	14	52	13.8	Large, pale Gray platelets
	18, M	*NBEAL2*		18	110	13.6	
	19, F	*NBEAL2*		15	115	12.7	
	36, F	*VWF*	VWF type 2B, AD	6	294	12.5	Macrothrombocyte
	47, F	*STIM1*	Immunodeficiency and thrombocytopenia, AR	5	153	11.9	Normo/macrothrombocyte
	43, F	*TUBB1*	Macrothrombocytopenia 1, AD	5	74	15	Macrothrombocyte
	46, M	*ACTB*	Thrombocytopenia 8 with dysmorphic features and developmental delay, AD	14	113	12.9	Macrothrombocyte
**Normothrombocyte**	50, F	*ANKRD26*	Thrombocytopenia 2, AD	8	140	11.5	Normo/macrothrombocyte
	3, M	*ETV6*	Thrombocytopenia 5, AD	29	182	10.1	Normotrombocyte
	4, F	*ETV6*		160	250	11.2	Normotrombocyte
	5, M	*FYB1*	Thrombocytopenia 3, AD	179	431	9.5	Normotrombocyte
	16, F	*ITGB3*	GT 2, AR	189	402	11.3	Normotrombocyte, platelet aggregation abnormality
	37, M	*GNE*	Thrombocytopenia 12 with or without myopathy, AR	8	65	8.7	Normotrombocyte
	38, M	*CDC42*	Takenouchi–Kosaki syndrome, AD	2	326	8.7	Normotrombocyte
	39, M	*IKZF1*	Familial immune thrombocytopenia, AD	1	18	10.9	Normotrombocyte
	40, F	*GFI1B*	Bleeding disorder, platelet-type 17, AD	7	58	11.1	Normotrombocyte
	41, F	*RBM8A*	TAR syndrome, AR	5	31	10.3	Normotrombocyte
	42, F	*RBM8A*		18	67	10.1	Normotrombocyte
	48, F	*MPL*	CAMT, AR	3	26	11.3	Normotrombocyte
	49, F	*MPL*		4	33	10.9	Normotrombocyte
	44, M	*RUNX1*	Familial platelet disorder with associated myeloid malignancy, AD	5	139	12.1	Normotrombocyte
	45, M	*FLI1*	Bleeding disorder, platelet-type 21, AD	6	126	10.6	Normotrombocyte
**Microthrombocyte**	20, F	*WAS*	Wiskott–Aldrich syndrome, X-linked recessive	8	61	5.1	Microthrombocyte
	21, M	*WAS*		8	48	7.6	Microthrombocyte
	22, M	*WAS*		8	53	6.9	Microthrombocyte
	23, M	*WAS*		5	45	6.8	Microthrombocyte
	24, M	*WAS*		5	36	5.8	Microthrombocyte
	25, M	*WAS*		3	54	6.3	Microthrombocyte
	26, M	*WAS*		23	98	5.3	Microthrombocyte
	27, M	*WAS*		11	35	7.2	Microthrombocyte
	28, M	*WAS*		8	58	4.8	Microthrombocyte
	29, M	*WAS*		3	42	7.6	Microthrombocyte
	30, M	*WAS*		5	35	8.8	Microthrombocyte
	31, M	*WAS*		8	44	6.7	Microthrombocyte
	32, M	*WAS*		2	78	6.7	Microthrombocyte
	33, M	*WAS*		11	54	7.6	Microthrombocyte
	34, M	*WAS*		5	54	6.9	Microthrombocyte
	35, M	*WAS*		16	52	8.2	Microthrombocyte

AD, autosomal dominant; AR, autosomal recessive; BSS, Bernard–Soulier syndrome; CAMT, congenital amegakaryocytic thrombocytopenia; F, female; GT, Glanzmann thrombasthenia; M, male; MPV, mean platelet volume; Plt, platelet; TAR, thrombocytopenia absent radii; VWF, von Willebrand factor.

### 3.4 Association between platelet levels and clinical bleeding severity

Separate analyses of the patient groups revealed distinct associations between platelet counts and bleeding severity. In patients with IT, there was a moderate, negative, and statistically significant correlation between platelet counts and bleeding severity (r = −0.56; *P* < 0.01). In contrast, patients with IPFD showed no statistically significant correlation (r = −0.23; *P* = .48). Additionally, patients with platelet counts below 10 × 10^3^/mm^3^ had a significantly higher risk of life-threatening bleeding episodes (odds ratio, 4.5; 95% CI, 2.1-7.8), underscoring the critical role of platelet counts in managing IPDs and emphasizing the necessity to differentiate between patient subgroups.

### 3.5 Platelet function analyses for selected patients

Among 50 patients in our cohort, platelet function testing was performed in 9 individuals with suspected receptor or granule-related platelet disorders. In 4 patients with BSS, flow cytometry revealed significantly reduced expression of CD42b, and LTA demonstrated absent ristocetin-induced aggregation with preserved responses to ADP and collagen. In 2 patients with GT, one showed markedly reduced CD41 expression by flow cytometry, while the other had impaired ADP-induced aggregation in LTA. In both cases, peripheral blood smears prepared from capillary blood revealed an absence of spontaneous platelet aggregation, further supporting the diagnosis of GT. Three patients clinically suspected of Gray platelet syndrome exhibited normal flow cytometry and aggregation responses; however, due to technical limitations, electron microscopy could not be performed to confirm alpha-granule deficiency. These functional findings, in combination with genetic results, supported the diagnostic classification in these complex cases.

### 3.6 IPDs: candidate genes and variants

We identified variants in 22 genes, including 12 autosomal dominant, 9 autosomal recessive, and 1 X-linked recessive (*WAS*; Table 3). The most implicated genes were *WAS* (16/50 variants), *MYH9* (5 variants), *GP1BA* (4 variants), and *NBEAL2* (3 variants). Notably, a female patient with *WAS*-related clinical features exhibited a unique X-inactivation pattern, offering new insights into the genetic mechanisms of IPDs. In addition, we detected variants in several known cancer predisposition genes (eg, *ETV6, RUNX1, ANKRD26,* and *IKZF1*), highlighting the importance of long-term surveillance and genetic counseling in IPDs with potential malignant transformation risk [19].

**Table 3:** Genetic variants and their characteristics identified in patients with inherited platelet disorders.

Patient	Gene (transcript)	Disease and inheritance	Variant— DNA level	Variant— protein level	Zygosity	Classification	Novelty
1	*ABCG5* (NM_022436.3)	Sitosterolemia, AR	c.904+5G>C	p.?	Hom.	Likely pathogenic	No
2			c.1336C>T	p.(Arg446Ter)	Hom.	Pathogenic	No
3	*ETV6* (NM_001987.5)	Thrombocytopenia 5, AD	c.1229A>G	p.(Glu410Gly)	Het.	VUS	No
4			c.776G>A	p.(Arg259Gln)	Het.	VUS	No
5	*FYB1* (NM_001465.6)	Thrombocytopenia 3, AD	c.1515+6G>A	p.?	Hom.	VUS	No
6	*MYH9* (NM_002473.6)	Macrothrombocytopenia and granulocyte inclusions with or without nephritis or sensorineural hearing loss, AD	c.283G>A	p.(Ala95Thr)	Het.	Pathogenic	No
7			c.5521G>A	p.(Glu1841Lys)	Het.	Pathogenic	No
8			c.719G>A	p.(Arg240His)	Het.	VUS	No
9			c.719G>A	p.(Arg240His)	Het.	VUS	No
10			c.4270G>A	p.(Asp1424Asn)	Het.	Pathogenic	No
11	*GP1BA* (NM_000173.7)	BSS, AR	c.378C>G	p.(Asn126Lys)	Hom.	Likely pathogenic	Yes
12			c.1591dup	p.(Leu531ProfsTer86)	Hom.	Likely pathogenic	Yes
13			c.1591dup	p.(Leu531ProfsTer86)	Hom.		
14			c.1525C>T	p.(Gln509Ter)	Hom.	Likely pathogenic	Yes
15	*ITGB3* (NM_000212.3)	Platelet-type 24, bleeding disorder, AD	c.1394C>G	p.(Ala465Gly)	Het.	VUS	Yes
16		GT 2, AR	c.1703G>A	p.(Cys568Tyr)	Hom.	Likely pathogenic	No
17	*NBEAL2* (NM_015175.3)	Gray platelet syndrome, AR	c.3796del	p.(His1266ThrfsTer9)	Hom.	Likely pathogenic	Yes
18			c.3796del	p.(His1266ThrfsTer9)	Hom.		
19			c.7279C>T	p.(Arg2427Cys)	Hom.	VUS	No
20	*WAS* (NM_000377.3)	Wiskott–Aldrich syndrome, XL	c.655G>T	p.(Gly219Ter)	Het.	Likely pathogenic	Yes
21			c.257G>A	p.(Arg86His)	Hem.	Pathogenic	No
22			c.1017del	p.(Gly340ValfsTer105)	Hem.	Pathogenic	No
23			c.778-2A>T	p.?	Hem.	Pathogenic	No
24			c.778-2A>T	p.?	Hem.		
25			c.371C>A	p.(Ala124Glu)	Hem.	Likely pathogenic	No
26			c.371C>A	p.(Ala124Glu)	Hem.		
27			Deletions of exons 1-2	p.?	Hem.	Likely pathogenic	Yes
28			c.10_11dup	p.(Pro5AlafsTer41)	Hem.	Pathogenic	No
29			c.631C>T	p.(Arg211Ter)	Hem.	Pathogenic	No
30			c.257G>A	p.(Arg86His)	Hem.	Pathogenic	No
31			c.371C>A	p.(Ala124Glu)	Hem.	Likely pathogenic	No
32			c.931+1G>C	p.?	Hem.	Likely pathogenic	No
33			c.1271dup	p.(Leu425ProfsTer70)	Hem.	Pathogenic	No
34			c.10_11dup	p.(Pro5AlafsTer41)	Hem.	Pathogenic	No
35			c.778-2A>T	p.?	Hem.	Pathogenic	No
36	*VWF* (NM_000552.5)	VWF type 2B, AD	c.3946G>A	p.(Val1316Met)	Het.	Pathogenic	No
37	*GNE* (NM_001128227.3)	Thrombocytopenia 12 with or without myopathy, AR	c.1768G>A	p.(Gly590Arg)	Hom.	Pathogenic	No
38	*CDC42* (NM_001791.4)	Takenouchi–Kosaki syndrome, AD	c.556C>T	p.(Arg186Cys)	Het.	Pathogenic	No
39	*IKZF1* (NM_006060.6)	Familial immune thrombocytopenia, AD	c.427C>T	p.(Arg143Trp)	Het.	Likely pathogenic	No
40	*GFI1B* (NM_001377304.1)	Bleeding disorder, platelet-type 17, AD	c.521C>T	p.(Thr174Ile)	Het.	VUS	No
41	*RBM8A* (NM_005105.5)	TAR syndrome, AR	c.-21G>A	p.?	Hom.	Pathogenic	No
42	*RBM8A* (NM_005105.5)		1.1 Mb deletion in 1q21.1 gene region	−	Het.	Pathogenic	No
43	*TUBB1* (NM_030773.4)	Macrothrombocytopenia 1, AD	c.704del	p.(Gly235AlafsTer2)	Het.	VUS	No
44	*RUNX1* (NM_001754.5)	Familial platelet disorder with associated myeloid malignancy, AD	c.1270T>G	p.(Ser424Ala)	Het.	VUS	No
45	*FLI1* (NM_002017.5)	Bleeding disorder, platelet-type 21, AD	c.152T>C	p.(Leu51Pro)	Het.	VUS	No
46	*ACTB* (NM_001101.5)	Thrombocytopenia 8 with dysmorphic features and developmental delay, AD	c.1011C>G	p.(Tyr337Ter)	Het.	Likely pathogenic	Yes
47	*STIM1* (NM_001382567.1)	Immunodeficiency 10, thrombocytopenia, AR	c.1987G>A	p.(Asp663Asn)	Hom.	VUS	No
48	*MPL* (NM_005373.3)	Amegakaryocytic thrombocytopenia, congenital 1, AR	c.805T>C	p.(Trp269Arg)	Hom.	VUS	No
49	*MPL* (NM_005373.3)		c.1511T>A	p.(Leu504His)	Hom.	VUS	Yes
50	*ANKRD26* (NM_014915.3)	Thrombocytopenia 2, AD	c.1846A>C	p.(Lys616Gln)	Het.	VUS	No

AD, autosomal dominant; AR, autosomal recessive; BSS, Bernard–Soulier syndrome; GT, Glanzmann thrombasthenia; Hem., hemizygous; Het., heterozygous; Hom., homozygous; TAR, thrombocytopenia absent radii; VUS, variant of uncertain significance; VWF, von Willebrand factor; XL, X-linked.

### 3.7 Novel variants in IPD-causing genes

This study identified 8 novel genetic variants, providing insight into their potential roles in IPDs. Among these, *GP1BA* (c.1591dup, c.1525C>T, and c.378C>G), *ITGB3* (c.1394C>G), *NBEAL2* (c.3796del), *WAS* (c.655G>T, deletions of exons 1-2), and *MPL* (c1511T>A) were notable for their association with severe thrombocytopenia and morphological platelet abnormalities. Furthermore, several rare mutations highlighted diverse contributions to bleeding phenotypes, immune dysregulation, and neurological and extremity abnormalities (Table 3).

Patient 13, who exhibited BSS due to a novel homozygous *GP1BA* variant, also presented with autism and intellectual disability, findings not typically associated with this disorder. Further genetic analysis identified a *de novo* heterozygous *FOXP1* frameshift variant (c.1240_1241del and p.Leu414Aspfs∗45), consistent with *FOXP1* syndrome, a neurodevelopmental disorder characterized by global developmental delay and autistic features. Interestingly, another unrelated patient (patient 12) carrying a similar *GP1BA* variant showed no neurological abnormalities, supporting the conclusion that neurodevelopmental features in patient 13 were attributable to *FOXP1* variant rather than *GP1BA*. This case illustrates the importance of comprehensive genetic evaluation in patients with complex, multisystemic presentations.

Each variant underwent rigorous evaluation, including computational modeling, population database comparisons, and literature reviews. Early evidence shows that these variants may cause disease affecting protein function or by interfering with the processes needed for making and functioning of platelets. These findings warrant further functional validation studies to elucidate their exact roles in the pathogenesis of IPDs.

### 3.8 Nonhematological findings and associated disorders

Nonhematological findings were prevalent among the cohort, reflecting the diverse clinical spectrum of IPDs. Patients with *CDC42* mutations presented with leukopenia, osteoporosis, hypotonia, and developmental delay. *IKZF1* mutations were associated with pancytopenia and immunodeficiency, while *ETV6* mutations were linked to hypogammaglobulinemia. Thrombocytopenia absent radii syndrome patients exhibited limb anomalies, including aplasia of the forearm bones (radii) and lower limb abnormalities that affected mobility. Some patients with *WAS* mutations showed allergic proctocolitis, eczema, and immunodeficiency features, with initial misdiagnoses of cow milk protein-induced allergic proctocolitis in certain cases. Clinical features of sitosterolemia due to *ABCG5* mutations included elevated plant sterol levels, xanthomas, premature atherosclerosis, hemolytic anemia (stomatocytosis), and macrothrombocytopenia. Additionally, the patient with *ACTB* mutation presented with keratoconus. These findings highlight the need for a multidisciplinary approach to managing IPDs.

### 3.9 Treatment

We implemented diverse therapeutic strategies following genetic diagnoses. We treated 2 patients with *ABCG5* mutations and sitosterolemia with ezetimibe to reduce cholesterol and plant sterol absorption, leading to the normalization of thrombocytopenia, peripheral smear findings, and hemolytic anemia. Sixteen patients underwent hematopoietic stem cell transplantation (HSCT), primarily for *WAS* (*n* = 12), followed by 1 *GNE*, 1 *IKZF1*, and 2 *MPL* cases. Among these, 4 of 16 (25%) patients succumbed posttransplantation due to graft failure (1 *GNE*) or infections (2 *WAS* and 1 *IKZF1*). However, 12 of 16 (75%) patients achieved disease-free survival, although 2 developed graft-versus-host disease, presenting as bronchiolitis obliterans. Nontransplanted patients received acute bleeding management, including platelet transfusions, tranexamic acid, von Willebrand factor (VWF) concentrates, and recombinant activated factor (F)VII. Transfusions were not routinely used for low platelet counts in the absence of bleeding. Where possible, leukocyte-depleted and/or irradiated platelet products were preferred to minimize the risk of alloimmunization and other transfusion-related complications. Recombinant activated FVII was administered in a limited number of patients with GT who experienced severe or refractory bleeding, particularly when platelet transfusions were ineffective or unavailable due to alloimmunization. Additionally, VWF concentrate was used in one patient with genetically confirmed VWF type 2B mutation presenting with life-threatening gastrointestinal bleeding. These agents were not used routinely but selectively, based on bleeding severity and clinical indication. Six patients who were given thrombopoietin receptor agonists (TPO-RAs) to prevent bleeding had fewer bleeding episodes. These patients had issues with the genes *RUNX1, FLI1, STIM1, MPL*, and *ANKRD26*. These approaches collectively improved both bleeding control and overall patient outcomes, emphasizing the importance of personalized strategies in managing IPDs.

## 4 Discussion

This study highlights the clinical and genetic spectrum of IPDs, providing insights into their diagnostic and therapeutic challenges. The identification of genetic mutations in our cohort is in accordance with previous findings in the literature, emphasizing the importance of genetic testing in patients who are frequently misdiagnosed with chronic ITP [5,6]. Before receiving an accurate genetic diagnosis, we initially followed most of our patients under an ITP diagnosis, a practice that reflects the diagnostic difficulties noted in the literature [20]. Our cohort’s high proportion of autosomal dominant inheritance (54.5%) underscores the importance of family bleeding history in the diagnostic process. This finding parallels observations in other global studies, further validating the role of genetic inheritance patterns in understanding IPDs [2,9].

While our findings underscore the growing relevance of genetic testing in IPD diagnostics, it is essential to emphasize that genetic analysis should not replace but rather complement comprehensive clinical assessment and conventional laboratory investigations, including peripheral blood smears, platelet morphology, and function testing. Functional assays, such as LTA, flow cytometry for platelet GP expression (eg, GPIb and GPIIb/IIIa), and, when indicated, electron microscopy for granule content, remain invaluable tools in characterizing platelet function and identifying receptor deficiencies [21]. Importantly, peripheral blood smear examination should be performed at the very beginning of the diagnostic workflow, as it provides critical morphologic clues, such as macrothrombocytopenia, granule abnormalities, or stomatocytosis, which can guide further targeted testing [12]. An integrated approach remains crucial, particularly in disorders where clinical phenotype or functional platelet defects guide initial suspicion.

Our study revealed distinct correlations between platelet counts and bleeding severity when evaluating IPD subgroups separately. In patients with IT, a statistically significant, moderate negative correlation (r = −.56; *P* = .0002) was demonstrated, corroborating earlier studies that indicate lower platelet counts are clearly associated with increased bleeding severity. In contrast, we observed no significant correlation in patients with IPFD (r = −.226; *P* = .48). This result aligns with existing literature, which emphasizes that bleeding risk in IPFD primarily depends on platelet functionality rather than platelet count alone [22]. These findings highlight the necessity of separately evaluating IPD subgroups to accurately interpret bleeding risks and guide effective clinical management. Our findings also underscore the importance of evaluating bleeding severity using standardized grading systems, which aids in both diagnosis and management [17]. Furthermore, high prevalence of anemia (80%) in our cohort, likely caused by chronic blood loss, is consistent with existing studies linking recurrent bleeding episodes to iron deficiency anemia in IPDs [23].

Gastrointestinal bleeding was notably prevalent among younger children carrying *WAS*, VWF type 2B mutations, or *GP1BA*-related BSS. Particularly in WAS, the combination of pronounced thrombocytopenia, platelet dysfunction, and immune dysregulation often manifests early on as proctocolitis and bloody diarrhea. Similarly, defects in VWF type 2B or *GP1BA* compromise platelet adhesion or function, elevating the risk of gastrointestinal hemorrhage. Recognizing these disease-specific patterns of bleeding is essential for instituting timely interventions and tailored management, ultimately improving outcomes in this vulnerable subset of pediatric patients.

The early onset of symptoms and the high frequencies of consanguinity in our cohort further underscore the significance of genetic research in regions with demographic characteristics that are comparable with those of our cohort. In addition, the fact that it takes longer to arrive at an accurate diagnosis highlights the necessity of doing more extensive genetic testing on individuals who experience recurrent bouts of thrombocytopenia and bleeding [6,9,24].

Peripheral smear examination remains a critical diagnostic tool, offering vital insights that directly influence clinical decisions and outcomes [4]. Findings such as stomatocytosis and macrothrombocytopenia provided essential clues for conditions like sitosterolemia, prompting targeted therapeutic interventions [12]. For instance, our findings showed a significant improvement in platelet counts, resolution of stomatocytosis, and normalization of hemolytic anemia in patients treated with ezetimibe. These results further reinforce prior evidence that ezetimibe is a cornerstone therapy for sitosterolemia, as detailed in the literature [12]. In addition, the unique morphologic findings in IPDs underline the role of peripheral smears as a cost-effective diagnostic modality.

We identified 8 novel variants in *GP1BA* (3 variants), *ITGB3, NBEAL2, WAS* (2 variants), and *MPL* genes, highlighting the diverse genetic underpinnings of IPDs [25]. These variants probably play a role in disease development by changing how proteins work or by interfering with important processes related to the production, activation, and clumping of platelets. For example, mutations in *GP1BA* and *ITGB3* may hinder platelet adhesion and aggregation, whereas those in *NBEAL2* and *WAS* are associated with more systemic hematological and immunologic complications [2,24]. Early evidence suggests that these factors are involved in the clinical traits seen. This highlights the need for further studies to confirm their harmful effects and understand the relationship between genetics and these traits. These findings highlight the importance of early genetic testing for better treatment plans, such as gene therapies and focused treatments, to help patients with IPDs [6].

Therapeutic interventions varied based on genetic diagnoses, with HSCT being the most effective treatment in severe cases, particularly in *WAS* patients [14,26,27]. This approach has also shown efficacy in *MPL-* and *IKZF1*-associated cases, as highlighted in other reports, emphasizing its broader applicability in managing severe IPDs [[28], [29], [30]]. Our cohort’s HSCT success rate (75%) is comparable with international data, emphasizing its efficacy [26]. However, complications such as graft failure and infections underline the need for careful patient selection and posttransplant monitoring.

Using TPO-RAs like eltrombopag or romiplostim helped to greatly lower bleeding episodes in our group. This shows that these treatments are effective in keeping platelet counts stable and reducing the symptoms of IPDs. This finding matches previous studies that highlight the usefulness of TPO-RAs for treating low platelet counts in different genetic types of IPDs, including those caused by mutations in *MPL*, *ANKRD26*, and other genes involved in making platelets [31,32]. By enhancing megakaryocyte proliferation and maturation, these agents help sustain platelet production, reducing the frequency and severity of bleeding episodes. Importantly, their use in our cohort was associated with an improved quality of life, as fewer patients required emergency interventions such as platelet transfusions or hospitalization for severe bleeding. Of 6 patients receiving TPO-RAs, most had initially been managed as chronic ITP. Genetic results subsequently confirmed diagnoses as IPD; however, clinicians chose to maintain TPO-RA therapy due to observed clinical benefits and limited bleeding episodes. Notably, our *MYH9* mutant patients rarely experienced severe bleeding and thus did not require TPO-RA therapy despite literature suggesting potential efficacy [31]. These findings emphasize the necessity of adopting personalized therapeutic strategies tailored to the molecular etiology and clinical severity of each case. TPO-RAs can be a good option for patients who cannot have invasive treatments like stem cell transplants. However, prolonged use necessitates long-term studies to evaluate potential side effects, such as thrombotic risks and bone marrow fibrosis. Overall, our findings reinforce the increasing agreement that TPO-RAs serve as a crucial component of IPD management, given careful monitoring and individualization according to patient-specific factors.

Although genetic testing has emerged as a powerful diagnostic tool, it should be interpreted within the clinical context and supported by functional assays when feasible. Especially in disorders such as BSS and GT, platelet GP analysis and aggregation studies continue to play a pivotal role in establishing diagnosis and guiding treatment. Thus, the optimal diagnostic strategy involves a synergistic approach that combines molecular, clinical, and laboratory findings.

### 4.1 Limitations

Our study has limitations, including its retrospective nature and relatively small sample size, which may limit the generalizability of findings. Furthermore, the absence of functional studies for novel variants leaves uncertainties about their pathogenic roles. Another limitation of our study is the lack of standardized bleeding assessment using a validated tool such as the ISTH Bleeding Assessment Tool. Due to the retrospective and multicenter design, bleeding symptoms were documented through structured clinical forms rather than formal scoring systems. While this allowed for consistent data collection from medical records, it limited our ability to perform detailed quantitative comparisons of bleeding severity across different IPD subtypes. Future prospective studies using validated bleeding assessment tools are needed to enable more precise genotype–phenotype correlations. Despite these limitations, the study provides valuable insights into the clinical and genetic characteristics of IPDs in a unique population.

## 5 Conclusion

This study highlights the importance of integrating genetic, clinical, and laboratory findings for the accurate diagnosis and management of IPDs. Early genetic diagnoses, multidisciplinary approaches, and personalized treatment strategies are essential for improving outcomes in this patient population. Future studies focusing on functional validation of novel mutations and long-term outcomes of therapeutic interventions are necessary to advance our understanding of IPDs.

## Declaration of AI and AI-Assisted Technologies in the Writing Process

During the preparation of this work the authors used ChatGPT 4o in order to make figure. After using this tool/service, the authors reviewed and edited the content as needed and take full responsibility for the content of the publication.
